# β‐amyloid impaired mitochondrial proteostasis through the impact on LONP1

**DOI:** 10.1002/alz.091488

**Published:** 2025-01-03

**Authors:** Wenzhang Wang

**Affiliations:** ^1^ Case Western Reserve Universit, CLEVELAND, OH USA

## Abstract

**Background:**

Mitochondrial dysfunction plays a critical role in the pathogenesis of Alzheimer’s disease (AD). Mitochondrial proteostasis regulated by chaperones and proteases in each compartment of mitochondria is critical for mitochondrial function, and it is suspected that mitochondrial proteostasis deficits may be involved in mitochondrial dysfunction in AD.

**Method:**

An unbiased screening of intraneuronal Aβ42 protein‐interactome was perfumed in AD cell culture. Mitochondrial protease activity and mitochondria functions were investigated in AD models in vitro and in vivo, while cognitive behaviors of AD transgenic mice were analyzed by memory capacity tests.

**Result:**

We identified LONP1, an ATP‐dependent protease in the matrix, as a top Aβ42 interacting mitochondrial protein through and found significantly decreased LONP1 expression and extensive mitochondrial proteostasis deficits in AD experimental models both in vitro and in vivo, as well as in the brain of AD patients. Impaired METTL3‐m^6^A signaling contributed at least in part to Aβ42‐induced LONP1 reduction. Moreover, Aβ42 interaction with LONP1 impaired the assembly and protease activity of LONP1 both in vitro and in vivo. Importantly, LONP1 knockdown caused mitochondrial proteostasis deficits and dysfunction in neurons, while restored expression of LONP1 in neurons expressing intracellular Aβ and in the brain of CRND8 APP transgenic mice rescued Aβ‐induced mitochondrial deficits and cognitive deficits.

**Conclusion:**

These results demonstrated a critical role of LONP1 in disturbed mitochondrial proteostasis and mitochondrial dysfunction in AD and revealed a novel mechanism underlying intracellular Aβ42‐induced mitochondrial toxicity through its impact on LONP1 and mitochondrial proteostasis.

